# Overall survival of Black patients with newly diagnosed multiple myeloma in the era of triplet and quadruplet therapies

**DOI:** 10.1038/s41408-026-01645-1

**Published:** 2026-10-07

**Authors:** Hamlet Gasoyan, Shahzad Raza, Michael B. Rothberg, Jeffrey D. Kovach, Nicholas J. Casacchia, Jason Valent, Faiz Anwer

**Affiliations:** 1 Center for Value-Based Care Research, Department of Internal Medicine and Geriatrics, Primary Care Institute, Cleveland Clinic, Cleveland, OH USA; 2 Cleveland Clinic Lerner College of Medicine of Case Western Reserve University, Cleveland, OH USA; 3 Department of Hematology and Medical Oncology, Taussig Cancer Center, Cleveland Clinic, Cleveland, OH USA; 4 Department of Quantitative Health Sciences, Lerner Research Institute, Cleveland Clinic, Cleveland, OH USA

**Keywords:** Myeloma, Epidemiology

Dear Editor,

Black individuals have a higher incidence of multiple myeloma (MM) and worse survival than White patients [1]. Growing evidence suggests that these survival differences are due to inequities in timely diagnosis and effective treatment utilization [2–4], given that Black patients generally exhibit equal or better survival than White patients after adjustment for key treatment-access barriers [2, 5]. Recent evidence also indicates that genomic features do not account for differences in MM risk across genetic ancestry groups [3]. An earlier Veterans Affairs (VA)-based study demonstrated that with equal access to myeloma therapy, Black patients have superior survival compared with White individuals [6]. However, it is unknown whether this finding holds in other care settings and in the era of triplet and quadruplet therapies. Here, we compare the overall survival of Black and White patients newly diagnosed with MM from 2017 to 2023 in a large civilian integrated health system. In a prior analysis of this cohort, we found no race-based differences in the use of triplet or quadruplet regimens within 1 year of diagnosis [7]. The present study extends that utilization-focused work by examining whether similar treatment patterns were accompanied by comparable survival outcomes.

This retrospective cohort study included adults 18 years of age or older with a new diagnosis of MM (defined as International Classification of Diseases [ICD]-O-3 code 9732/3 and histologically proven active MM in the Myeloma Patient Registry) between January 1, 2017, and December 31, 2023. Treatment locations included Taussig Cancer Center at the main campus of Cleveland Clinic and its Ohio and Florida regional hospitals. Patients were excluded if they received an initial diagnosis at the Cleveland Clinic but completed treatment elsewhere, if they received an initial diagnosis elsewhere and presented at Cleveland Clinic with disease recurrence or persistence, or if they were missing insurance information, area deprivation index (ADI), or baseline estimated glomerular filtration rate (eGFR) lab values, as missingness on those variables was indicative of incomplete records based on data diagnostics and manual chart review [7].

The primary outcome of this study was patients’ overall survival (OS), captured through April 30, 2026, using the Cleveland Clinic electronic health record, including linked state and federal death records. The primary predictor variable was race, classified as White, Black, or other, based on fixed categories from the EHR record. Other sociodemographic variables included sex, age at diagnosis, insurance type, ADI [8], and Rural-Urban Commuting Area, each captured using the data point closest to the initial diagnosis. Clinical and treatment access-related variables included International Staging System (ISS) stage at diagnosis, Eastern Cooperative Oncology Group (ECOG) performance status, baseline estimated glomerular filtration rate (eGFR), Charlson Comorbidity Index (CCI), high-risk cytogenetics based on IMS/IMWG 2025 recommendations [9], receipt of any triplet or quadruplet regimen within a year of diagnosis (captured using an earlier described algorithm) [10], and receipt of stem cell transplant during study follow-up.

We compared baseline characteristics using Pearson’s chi-squared test, Fisher’s exact test for count data with a simulated *p*-value (based on 2000 replicates), and the Kruskal-Wallis rank sum test, as appropriate. Probability of 5-year overall survival was estimated using the Kaplan–Meier method. We also present the adjusted probability of 5-year overall survival for Black and White patients using overlap weighting, which balanced baseline characteristics between these groups, including ISS, ECOG performance status, baseline eGFR, CCI, high-risk cytogenetics, sex, age at diagnosis, insurance type, ADI, Rural-Urban Commuting Area, and year of diagnosis [11]. Overlap weighting is a propensity score method that emphasizes patients with the greatest covariate overlap between comparator groups, thereby improving balance between groups [11].

A multivariable Cox regression model examined the association of race with 5-year all-cause mortality. The Cox model was adjusted for receipt of either a triplet or quadruplet regimen, receipt of stem cell transplant, ISS, ECOG performance status, baseline eGFR, CCI, high-risk cytogenetics, sex, age at diagnosis, insurance type, ADI, Rural-Urban Commuting Area, and year of diagnosis. Missing data within variables were grouped under an “unknown” category; imputation for missing data was not used. Patients were right-censored either at the end of the study period (April 30, 2026), 5 years after their initial diagnosis date, or at the end of available follow-up in the EHR, whichever occurred first. In sensitivity analysis, we restricted the cohort to those who received at least 1 medication commonly used for MM induction therapy [10]. All statistical testing was two-tailed with an alpha of 0.05 used to determine statistical significance. All analyses were conducted in R, version 4.4.1 (R Foundation for Statistical Computing, Vienna, Austria). The study was approved by the Cleveland Clinic Institutional Review Board as minimal risk research using data collected for routine clinical practice, for which the requirement for informed consent was waived (approval number: 22-1181).

We identified 1209 patients, 54.1% male, 74.2% White, 22.7% Black, and 3.1% other races. Mean (SD) age at diagnosis was 67.3 (11.0). At diagnosis, 27.8% of patients were ISS stage 1, 66.3% had an ECOG performance status of 0–1, 60.3% had a baseline eGFR ≥60, and 51.0% had a CCI of ≤2. Within one year, 686 patients (56.7% of the cohort) received either triplet or quadruplet therapy, including 55.9% of White and 58.2% of Black patients. Table 1 presents the distribution of other socio-demographic and clinical variables by race. The distribution of baseline covariates between White and Black groups was precisely balanced (standardized differences = 0) after overlap weighting.

**Table 1:** Characteristics of patients diagnosed with multiple myeloma between 2017 and 2023 by race.

Characteristic, No (%)	Overall (*N* = 1209)	White (*n* = 897)	Black (*n* = 275)	Other (*n* = 37)	*P*-value^a^
Sex					0.09
Male	654 (54.1%)	501 (55.9%)	133 (48.4%)	20 (54.1%)	
Female	555 (45.9%)	396 (44.1%)	142 (51.6%)	17 (45.9%)	
Age at diagnosis, mean (SD)	67.3 (11.0)	67.8 (10.8)	66.0 (11.6)	64.8 (11.9)	0.04
Insurance type					<0.001
Private	411 (34.0%)	322 (35.9%)	74 (26.9%)	15 (40.5%)	
Traditional medicare	354 (29.3%)	292 (32.6%)	57 (20.7%)	5 (13.5%)	
Medicare advantage	308 (25.5%)	222 (24.7%)	75 (27.3%)	11 (29.7%)	
Medicaid	90 (7.4%)	41 (4.6%)	46 (16.7%)	3 (8.1%)	
Dual eligible	37 (3.1%)	14 (1.6%)	21 (7.6%)	2 (5.4%)	
Self-pay/Other	9 (0.7%)	6 (0.7%)	2 (0.7%)	1 (2.7%)	
Treatment facility					<0.001
Taussig Cancer Center	678 (56.1%)	535 (59.6%)	133 (48.4%)	10 (27.0%)	
Florida hospitals	317 (26.2%)	222 (24.7%)	72 (26.2%)	23 (62.2%)	
Ohio regional hospitals	214 (17.7%)	140 (15.6%)	70 (25.5%)	4 (10.8%)	
Area Deprivation Index quartile^b^					<0.001
1–25	199 (16.5%)	161 (17.9%)	23 (8.4%)	15 (40.5%)	
26–50	304 (25.1%)	263 (29.3%)	34 (12.4%)	7 (18.9%)	
51–75	338 (28.0%)	282 (31.4%)	48 (17.5%)	8 (21.6%)	
76–100	368 (30.4%)	191 (21.3%)	170 (61.8%)	7 (18.9%)	
Year of initial diagnosis					>0.9
2017	173 (14.3%)	135 (15.1%)	33 (12.0%)	5 (13.5%)	
2018	159 (13.2%)	113 (12.6%)	43 (15.6%)	3 (8.1%)	
2019	163 (13.5%)	121 (13.5%)	37 (13.5%)	5 (13.5%)	
2020	181 (15.0%)	132 (14.7%)	42 (15.3%)	7 (18.9%)	
2021	185 (15.3%)	138 (15.4%)	43 (15.6%)	4 (10.8%)	
2022	213 (17.6%)	155 (17.3%)	51 (18.5%)	7 (18.9%)	
2023	135 (11.2%)	103 (11.5%)	26 (9.5%)	6 (16.2%)	
Urbanicity of primary residence					<0.001
Metropolitan	1,064 (88.0%)	760 (84.7%)	270 (98.2%)	34 (91.9%)	
Micropolitan	89 (7.4%)	82 (9.1%)	4 (1.5%)	3 (8.1%)	
Small town or rural	56 (4.6%)	55 (6.1%)	1 (0.4%)	0 (0.0%)	
Charlson Comorbidity Index					<0.001
≤2	617 (51.0%)	495 (55.2%)	104 (37.8%)	18 (48.6%)	
3-4	212 (17.5%)	163 (18.2%)	42 (15.3%)	7 (18.9%)	
≥5	380 (31.4%)	239 (26.6%)	129 (46.9%)	12 (32.4%)	
Baseline eGFR					<0.001
≥60	729 (60.3%)	570 (63.5%)	134 (48.7%)	25 (67.6%)	
<60	480 (39.7%)	327 (36.5%)	141 (51.3%)	12 (32.4%)	
ECOG performance status					0.007
0-1	801 (66.3%)	614 (68.5%)	159 (57.8%)	28 (75.7%)	
2	166 (13.7%)	112 (12.5%)	53 (19.3%)	1 (2.7%)	
3-4	97 (8.0%)	66 (7.4%)	28 (10.2%)	3 (8.1%)	
Unknown	145 (12.0%)	105 (11.7%)	35 (12.7%)	5 (13.5%)	
ISS staging					0.4
Stage 1	336 (27.8%)	247 (27.5%)	75 (27.3%)	14 (37.8%)	
Stage 2	308 (25.5%)	232 (25.9%)	66 (24.0%)	10 (27.0%)	
Stage 3	311 (25.7%)	225 (25.1%)	81 (29.5%)	5 (13.5%)	
Unknown	254 (21.0%)	193 (21.5%)	53 (19.3%)	8 (21.6%)	
High risk cytogenetics					<0.001
Yes	108 (8.9%)	84 (9.4%)	18 (6.5%)	6 (16.2%)	
No	477 (39.5%)	332 (37.0%)	135 (49.1%)	10 (27.0%)	
Unknown	624 (51.6%)	481 (53.6%)	122 (44.4%)	21 (56.8%)	
Triplet or quadruplet therapy within 1 year					0.3
Yes	686 (56.7%)	501 (55.9%)	160 (58.2%)	25 (67.6%)	
No	523 (43.3%)	396 (44.1%)	115 (41.8%)	12 (32.4%)	
Stem cell transplant during follow-up					<0.001
Yes	416 (34.4%)	345 (38.5%)	62 (22.5%)	9 (24.3%)	
No	793 (65.6%)	552 (61.5%)	213 (77.5%)	28 (75.7%)	

*ECOG* Eastern Cooperative Oncology Group, *eGFR* estimated glomerular filtration rate.

^a^Based on Pearson’s chi-squared test, Fisher’s exact test for count data with simulated *p*-value (based on 2000 replicates), or Kruskal-Wallis rank sum test.

^b^Area Deprivation Index scores range from 1 to 100 with higher scores indicating greater socioeconomic disadvantage.

The probability of 5-year OS in the overall cohort was 61.3% (95% CI, 58.2–64.7%). Figure 1 presents the unadjusted (panel A) and overlap weighted (panel B) Kaplan–Meier curves of 5-year survival probability by race. In the overlap weighted analysis, the probability of 5-year OS was 58.3% (95% CI, 53.2–63.8%) in White and 59.8% (95% CI, 52.9–67.6%) in Black patients.

**Fig. 1:**
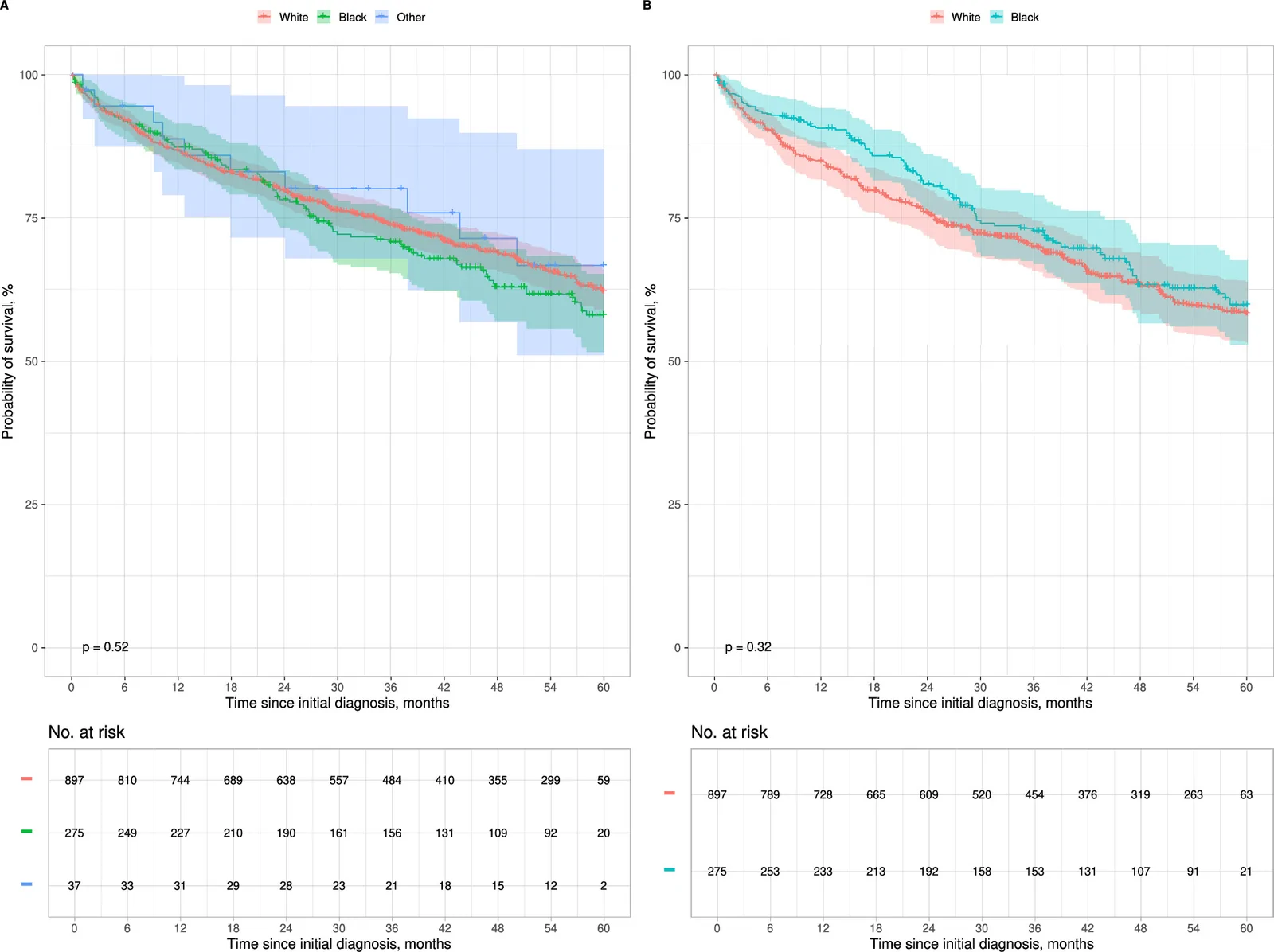
Kaplan–Meier curves of survival probability. Unadjusted (**a**) and adjusted (**b**) probability of 5-year overall survival by race. We identified adult patients newly diagnosed with multiple myeloma between January 1, 2017, and December 31, 2023. Patients were right-censored either at the end of the study period (April 30, 2026), 5 years after their initial diagnosis date, or end of available follow-up, whichever occurred first. Adjusted probability of 5-year overall survival is based on overlap weighted analysis, which balanced baseline characteristics between groups, including International Staging System stage at diagnosis, ECOG performance status, baseline estimated glomerular filtration rate, Charlson Comorbidity Index, high-risk cytogenetics, sex, age at diagnosis, insurance type, Area Deprivation Index, urbanicity of primary residence, and year of diagnosis. Patients with other races were not included in the overlap-weighted analysis due to small sample size.

In the multivariable Cox regression model for all-cause mortality at 5 years, adjusted hazard ratio (aHR) for death for Black patients vs. White was 0.73 (95% CI, 0.55–0.97). The results of the sensitivity analysis supported the main findings (aHR for Black vs. White race, 0.74, 95% CI, 0.55–1.00).

This study has some limitations. It used data from a single large integrated health system in Ohio and Florida, which may limit its generalizability. Although the use of upfront triplet or quadruplet therapy was similar across racial groups, stem cell transplant was used less frequently among Black patients compared to White patients, suggesting variability in utilization of available treatment modalities. This is consistent with prior literature on stem cell transplant use [12], and highlights a research gap regarding barriers to stem cell transplant utilization that could be further explored through qualitative research approaches. This study also did not capture the receipt of maintenance therapy or further lines of therapy, and their impact of survival outcomes could not be examined. Because the use and composition of FISH testing varied over the study period, our data on high-risk cytogenetic abnormalities were incomplete; therefore, the lower incidence of high-risk cytogenetics observed among Black patients compared with White patients in this study should be interpreted with caution. We were also unable to assess reasons for treatment refusal and may have missed some treatments received outside the health system. Our study also has important strengths, including a large and diverse cohort of patients with confirmed MM, longitudinal treatment data spanning the adoption of novel induction therapies, balanced real-world use of triplet or quadruplet therapy among White and Black patients, a rich set of sociodemographic and clinical covariates, and follow-up for overall survival through 2026.

In this large and diverse cohort of patients with MM who had equal access to triplet or quadruplet therapies, Black patients had overall survival at 5 years comparable to that of White patients in unadjusted and overlap weighted analyses and a lower risk of all-cause mortality at 5 years after adjustment for relevant sociodemographic, clinical, and treatment-related variables. These findings are consistent with those of a prior VA study that included patients who were diagnosed with MM from 2000 to 2017 [6]. Our results further indicate that health equity initiatives aimed at improving access to multiple myeloma care among minority patients have the promise of addressing race-based disparities in survival [5, 13]. This issue is particularly timely and important as treatments for MM become more efficacious but also more expensive, and vigilance of policymakers and clinicians is needed to make sure that all patients with MM benefit from treatment advances equally [13–15].

## Data Availability

The dataset generated during the current study is not publicly available to preserve patient confidentiality. However, the dataset is available from the corresponding author to academic investigators following receipt of a signed data-sharing agreement and after reviewing the study protocol (approved by a local institutional review board or research ethics committee), statistical analysis plan, and publication plan. The Cleveland Clinic Institutional Review Board and the Law Department must approve the request before sharing the de-identified data. The corresponding author will respond to these requests within 2 months of receipt.
